# Supplementary material for: Prediction of stimulus-independent and task-unrelated thought from functional brain networks
Source: Nat Commun. 2021 Mar 19;12:1793. doi: 10.1038/s41467-021-22027-0 (PMC7979817; doi:10.1038/s41467-021-22027-0)
Supplement: Supplementary file 1 — Supplementary Information [file 41467_2021_22027_MOESM1_ESM.pdf]

Supplementary Information

Prediction of stimulus-independent and task-unrelated thought from functional brain networks

Kucyi et al.

SITUT-CPM vs. SA-CPM

a

Network Overlap

	**SITUT-CPM** Positive	**SITUT-CPM** Negative
**SA-CPM** Positive	0% overlap *P* = 1	0.72% overlap *P* = 1
**SA-CPM** Negative	2.3% overlap *P* = 0.30	0% overlap *P* = 1

b

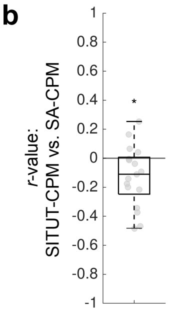

SITUT-CPM vs. Creativity-CPM

c

Network Overlap

	**SITUT-CPM** Positive	**SITUT-CPM** Negative
**Cr-CPM** Positive	0.78% overlap *P* = 0.22	1.43% overlap *P* = 0.031
**Cr-CPM** Negative	2.3% overlap *P* = 0.072	5.04% overlap *P* = 4.8×10^{-5}

d

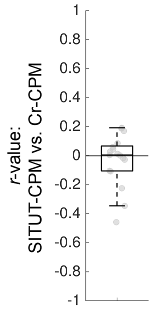

Figure S1. Comparison of the SITUT-CPM with other CPMs previously defined in published studies. **a)** Percent overlap between edges contributing to the SITUT-CPM and the Sustained Attention (SA) CPM. *P* values indicate significance of overlap based on the hypergeometric cumulative density function. **b)** Trial-by-trial correlation between SITUT-CPM and SA-CPM strength within each subject; at the group level (n=17), the mean correlation was significantly lesser than zero (*P*=0.031, two-tailed, Wilcoxon signed rank test). **c)** Same as a) but for overlap between the SITUT-CPM and the Creativity CPM. **d)** Trial-by-trial correlation between SITUT-CPM and Creativity CPM strength within each subject; at the group level (n=17), the mean correlation was not significantly different from zero (*P*=0.94, two-tailed, Wilcoxon signed rank test). In **b)** and **d)**, central mark indicates median, edges of box indicate 25th and 75th percentile, and whiskers extend to most extreme datapoints not considered to be outliers (defined as ± 2.7 SD). Source data are provided as a Source Data file.

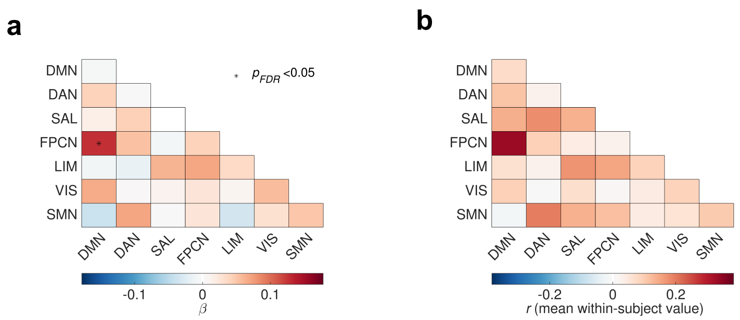

Figure S2. a) Coefficient values from linear mixed effects model analyses, using the mean functional connectivity across all region pairs for each network pair shown as a predictor of trial-wise SITUT rating. Among all network pairs within the Yeo-Krienen 7-network atlas, only DMN-FPCN was significantly associated with SITUT at the group-level when correcting for all network pair comparisons ($F_{1,612}$ = 10.2, P_{FDR} = 0.044, two-sided; *F* test on linear mixed effects model). Source data are provided as a Source Data file. **b)** Mean within-subject correlation between network-pair functional connectivity and SITUT-CPM strength across all trials. SITUT-CPM strength showed greatest correlation with DMN-FPCN connectivity (mean within-subject *r* = 0.33).

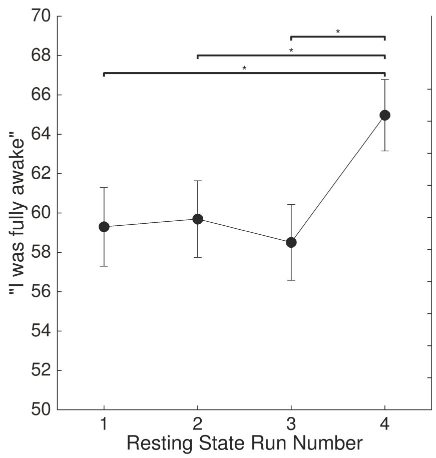

Figure S3. Subjective ratings of wakefulness in the Leipzig dataset. Post-run subjective ratings for the item “I was fully awake” showed a significant interaction with run number ($F_{1,502.39}$ = 6.25, P = 0.013; F test on linear mixed effects model; participants included in analyses: n=164 for run 1; n=164 for run 2; n=164 for run 3; n=162 for run 4), while post-hoc tests revealed significant increases in wakefulness rating for run 4 relative to runs 1, 2 and 3 (P_{FDR} = 0.039, 0.025, and 0.0022 respectively; Wilcoxon signed rank tests, two-sided). This increase in subjective wakefulness, specific to the last run, could have been due to the fact that participants were instructed when the final run was going to begin (M. Lauckner and D. Margulies, personal communication). Changes in wakefulness were not likely to be a source of variability SITUT-CPM strength, which exhibited significant changes among runs 1-3 (**Fig. 7**) while self-reported wakefulness remained stable. Source data are provided as a Source Data file.

Supplementary Table 1. Associations between CPM-based network strengths (for SITUT-CPM and SA-CPM) and ADHD symptom severity, as assessed based on the Kiddie Schedule for Affective Disorders and Schizophrenia (K-SADS) with separate scores for current (items rated based on the past 6 months) and diagnostic (items rated based on the past in general) symptoms. Spearman's rank correlation coefficients are shown uncorrected *P* values (two-sided). Source data are provided as a Source Data file.

	**Current Hyperactivity**	**Diagnostic Hyperactivity**	**Current Inattention**	**Diagnostic Inattention**
**SITUT-CPM**	ρ = 0.13<br>*P* = 0.36	ρ = 0.17<br>*P* = 0.25	ρ = 0.11<br>*P* = 0.44	ρ = 0.23<br>*P* = 0.10
**SA-CPM**	ρ = 0.16<br>*P* = 0.27	ρ = 0.12<br>*P* = 0.41	ρ = 0.17<br>*P* = 0.23	ρ = 0.35<br>*P* = 0.013
