# Supplementary material for: Prediction of stimulus-independent and task-unrelated thought from functional brain networks
Source: Nat Commun. 2021 Mar 19;12:1793. doi: 10.1038/s41467-021-22027-0 (PMC7979817; doi:10.1038/s41467-021-22027-0)
Supplement: Supplementary file 3 — Reporting Summary [file 41467_2021_22027_MOESM3_ESM.pdf]

nature research

Corresponding author(s): Aaron Kucyi

Last updated by author(s): Feb 1, 2021

Reporting Summary

Nature Research wishes to improve the reproducibility of the work that we publish. This form provides structure for consistency and transparency in reporting. For further information on Nature Research policies, see our Editorial Policies and the Editorial Policy Checklist.

Statistics

For all statistical analyses, confirm that the following items are present in the figure legend, table legend, main text, or Methods section.

n/a	Confirmed	
☐	☒	The exact sample size (*n*) for each experimental group/condition, given as a discrete number and unit of measurement
☐	☒	A statement on whether measurements were taken from distinct samples or whether the same sample was measured repeatedly
☐	☒	The statistical test(s) used AND whether they are one- or two-sided<br>*Only common tests should be described solely by name; describe more complex techniques in the Methods section.*
☐	☒	A description of all covariates tested
☐	☒	A description of any assumptions or corrections, such as tests of normality and adjustment for multiple comparisons
☐	☒	A full description of the statistical parameters including central tendency (e.g. means) or other basic estimates (e.g. regression coefficient) AND variation (e.g. standard deviation) or associated estimates of uncertainty (e.g. confidence intervals)
☐	☒	For null hypothesis testing, the test statistic (e.g. F, t, r) with confidence intervals, effect sizes, degrees of freedom and P value noted<br>*Give P values as exact values whenever suitable.*
☒	☐	For Bayesian analysis, information on the choice of priors and Markov chain Monte Carlo settings
☒	☐	For hierarchical and complex designs, identification of the appropriate level for tests and full reporting of outcomes
☐	☒	Estimates of effect sizes (e.g. Cohen's *d*, Pearson's *r*), indicating how they were calculated

Our web collection on statistics for biologists contains articles on many of the points above.

Software and code

Policy information about availability of computer code

Data collection	MGH study: Psychophysics Toolbox-3 used for presentation of task stimuli
Data analysis	Custom Matlab code for connectome-based predictive modeling analyses are publicly available on Github: https://github.com/swglab/CPM_CONN

For manuscripts utilizing custom algorithms or software that are central to the research but not yet described in published literature, software must be made available to editors and reviewers. We strongly encourage code deposition in a community repository (e.g. GitHub). See the Nature Research guidelines for submitting code & software for further information.

Data

Policy information about availability of data

All manuscripts must include a data availability statement. This statement should provide the following information, where applicable:

- Accession codes, unique identifiers, or web links for publicly available datasets
- A list of figures that have associated raw data
- A description of any restrictions on data availability

Data from the Brain Genomics Superstruct (https://www.neuroinfo.org/gsp) and Leipzig (https://www.nitrc.org/projects/mpilmbb) datasets are publicly available. For the MGH and MIT datasets, original functional and structural MR images can be made available upon reasonable request to the authors with mandatory ethics approval and required data use agreements with Massachusetts General Hospital and/or Massachusetts Institutes of Technology.

Field-specific reporting

Please select the one below that is the best fit for your research. If you are not sure, read the appropriate sections before making your selection.

- [x] Life sciences
- [ ] Behavioural & social sciences
- [ ] Ecological, evolutionary & environmental sciences

For a reference copy of the document with all sections, see nature.com/documents/nr-reporting-summary-flat.pdf

Life sciences study design

All studies must disclose on these points even when the disclosure is negative.

Sample size

Final samples:

MGH study (Dataset 1): n=17 healthy adults (within-group CPM analysis), n=28 healthy adults (group comparison analyses) and n=20 ADHD adults (all ADHD analyses)
- No sample size calculation was performed due to the novelty of our analysis approach. The sample sizes of individuals and trials per individual were chosen based on prior fMRI studies that have linked fMRI activation and connectivity with online self-report measures assessed with experience sampling. Past studies suggest that n=17 with 36 trials per subject would likely be sufficient for detecting statistically significant relationships at the group level; here we extended this approach to a novel connectome-based predictive modeling framework and hypothesized that a similar sample size would yield significant results.

Superstruct: n=911
- No sample size calculation was performed; sample size was determined based on publicly available data (i.e., the number of participants in the Superstruct dataset that underwent resting state fMRI with acceptable head motion and also completed the daydreaming frequency scale). This dataset was used for external validation of our model developed based on the MGH study. Compared to prior work on associations between resting state functional connectivity and individual differences in self-report outcomes within healthy samples, the sample size was considered suitable for our external validation analysis.

Leipzig: n=144 (sample size determined based on publicly available data)
- No sample size calculation was performed; sample size was determined based on publicly available data (i.e., the number of participants in the Leipzig dataset that underwent resting state fMRI with acceptable head motion and completed the Mind Wandering Spontaneous/Deliberate questionnaire). This dataset was used for external validation of our model developed based on the MGH study. Compared to prior work on associations between resting state functional connectivity and individual differences in self-report outcomes within healthy samples, the sample size was considered suitable for our external validation analysis.

MIT: n=49 (no sample size calculation performed)
- No sample size calculation was performed; sample size was determined based on available data (i.e., the number of participants in the MIT dataset that underwent resting state fMRI with acceptable head motion and completed the Mind Wandering Questionnaire). This dataset was used for external validation of our model developed based on the MGH study. Compared to prior work on associations between resting state functional connectivity and individual differences in self-report outcomes within clinical samples, the sample size was considered suitable for our external validation analysis.

Data exclusions

Participants with high levels of head motion during fMRI scanning were excluded because head motion is known to obscure estimates of functional connectivity. In all datasets, articipants with mean overall frame-wise displacement (FD) of >0.15 mm (based on the Jenkinson method) were excluded from analyses. In the Leipzig dataset, motion exclusion threshold was based on the mean across four fMRI runs, and we additionally excluded individual runs showing an FD value exceeding the 75th percentile plus 1.5 times interquartile range.

Replication

Out-of-sample validation was performed in 4 datasets

Randomization

Not relevant to this study, as no intervention effects were assessed in the analyses. Patients with ADHD were diagnosed with pre-established criteria.

Blinding

Investigators were not blinded. No intervention effects were assessed in the analyses. However, it is possible that the lack of blinding may have impacted the fMRI markers examined in this study.

Reporting for specific materials, systems and methods

We require information from authors about some types of materials, experimental systems and methods used in many studies. Here, indicate whether each material, system or method listed is relevant to your study. If you are not sure if a list item applies to your research, read the appropriate section before selecting a response.

Materials & experimental systems

n/a	Involved in the study
☒	☐ Antibodies
☒	☐ Eukaryotic cell lines
☒	☐ Palaeontology and archaeology
☒	☐ Animals and other organisms
☐	☒ Human research participants
☒	☐ Clinical data
☒	☐ Dual use research of concern

Methods

n/a	Involved in the study
☒	☐ ChIP-seq
☒	☐ Flow cytometry
☐	☒ MRI-based neuroimaging

Human research participants

Policy information about studies involving human research participants

Population characteristics	MGH: age range (18-35), males and females, healthy adults and adults diagnosed with ADHD<br>Superstruct: age range (18-35), males and females, healthy adults<br>Leipzig: age range (20-75), males and females, healthy adults<br>MIT: age range (18-45), males and females, adults diagnosed with ADHD
Recruitment	In all datasets, participants were recruited via online and poster advertisements. It is therefore possible that self-selection biases (e.g. selective recruitment of individuals who tend to search online for studies to participate in) may be present. These biases are unlikely to have strongly impacted the results, as the experience of stimulus-independent and task-unrelated thought (our main outcome measure) is not known to be associated with such biases.
Ethics oversight	Partners Human Research Institutional Review Board, Ethics committee at the medical faculty of the University of Leipzig, (097/15-ff), MIT institutional review board, Harvard University Committee on the Use of Human Subjects in Research

Note that full information on the approval of the study protocol must also be provided in the manuscript.

Magnetic resonance imaging

Experimental design

Design type	Task and resting state
Design specifications	36 trials (thought probes) per subject in MGH dataset
Behavioral performance measures	MGH dataset:<br>Self-report (scales ranging from 0-100)<br>Reaction time variability (computed as absolute deviation from mean for each trial)

Acquisition

Imaging type(s)	Functional and structural (all datasets)
Field strength	3 Tesla (all datasets)
Sequence & imaging parameters	MGH:<br>Functional: multiband acceleration factor (4), TR (1.08 sec), TE (30 sec), flip angle (60°), slice number (68), resolution (2 mm isotropic)<br>Structural: TR (2.53 sec), TE (1.15 ms), flip angle (7°), TI (1.1 sec), resolution (1 mm isotropic)<br><br>Superstruct:<br>Functional: duration (6 min 12 sec), TR (3.0 sec), TE (30 msec), flip angle (85°), slice number (47), resolution (3.0 mm isotropic)<br>Structural: duration (2 min 12 sec), TR (2.2 sec), TE (1.5/3.4/5.2/7.0 msec), flip angle (7°), TI (1.1 sec), slice number (144), resolution (1.2 mm isotropic)<br><br>Leipzig:<br>Functional: duration (15 min 30 sec), multiband acceleration factor (4), TR (1.4 sec), TE (39.4 msec), flip angle (69°), echo spacing (0.67 msec), slice number (64), resolution (2.3 mm isotropic)<br>Structural: duration (8.22 min), TR (5 sec), TE (2.92 msec), flip angle 1/2 (4/5°), TI 1/2 (700/2500 msec), slice number (176), resolution (1.0 mm isotropic)<br><br>MIT:<br>Functional: duration (7 min), multiband acceleration factor (6), TR (1.0 sec), TE (38 msec), flip angle (61°), slice number (66), resolution (2.0 x 2.0 x 2.2 mm isotropic)

	Structural: TR (2.53 sec), TE (2.3 msec), flip angle (9°), TI (0.9 sec), slice number (176), resolution (1.0 mm isotropic)
Area of acquisition	Whole brain

Diffusion MRI ☐ Used ☒ Not used

Preprocessing

Preprocessing software	CONN toolbox (version 19c) and SPM12 in Matlab R2019a (Mathworks Inc., Natick, MA)
Normalization	Linear realignment
Normalization template	ICBM152
Noise and artifact removal	Linear regression of the following parameters from each voxel: a) 5 noise components each from minimally-eroded WM and CSF (one-voxel binary erosion of voxels with values above 50% in posterior probability maps), respectively, based on aCompCor procedures; b) 12 motion parameters (3 translation, 3 rotation, and associated first-order derivatives); c) all outlier frames identified within participant; and d) linear BOLD signal trend within session. In a separate step after nuisance regression, data were then temporally filtered with a band-pass of 0.01-0.1 Hz.
Volume censoring	CONN toolbox: frame-wise displacement > 0.9 mm or global BOLD signal change > 5 SD

Statistical modeling & inference

Model type and settings	Connectome-based predictive modeling
Effect(s) tested	Prediction of self-report scores (correlation between observed and predicted scores)

Specify type of analysis: ☐ Whole brain ☒ ROI-based ☐ Both

Anatomical location(s): Shen and Schaefer atlases

Statistic type for inference (See Eklund et al. 2016)

All of our analyses were ROI-based; as such voxel-wise analyses were not performed. Inferences were based on the following tests:

MGH study (Figures 1 and 3): Wilcoxon signed rank test to compare predictions with permuted predictions at the group-level; Wilcoxon rank sum test to compare CPM strength in ADHD versus HC participants.

Superstruct and Leipzig datasets (Figures 4 and 5): Spearman correlation between predicted and observed behavioral outcome

MIT dataset (Figure 6): Wilcoxon rank sum test (to compare CPM strength between ADHD subgroups)

Leipzig dataset (Figure 7): F-test on linear mixed effects model with post-hoc Wilcoxon rank sum tests.

Correction

False-discovery rate correction was applied to the Leipzig dataset analysis (Figure 7), where multiple different pairs of resting state fMRI runs were being compared with one another. For other analyses presented, no correction was performed, as multiple tests were not performed.

Models & analysis

n/a	Involved in the study
☐	☒ Functional and/or effective connectivity
☒	☐ Graph analysis
☐	☒ Multivariate modeling or predictive analysis

Functional and/or effective connectivity	Pearson correlation
Multivariate modeling and predictive analysis	Independent variables: pair-wise functional connectivity between atlas regions<br>Feature extraction and dimension reduction: retained top connections (p<0.01, uncorrected) that were correlated with the dependent variable within training dataset<br>Evaluation metrics: Pearson or Spearman correlation between observations and predictions, MSE
